# Assessment of Workflow Feature Selection on Forest LAI Prediction with Sentinel-2A MSI, Landsat 7 ETM+ and Landsat 8 OLI

**DOI:** 10.3390/rs12060915

**Published:** 2020-03-12

**Authors:** Benjamin Brede, Jochem Verrelst, Jean-Philippe Gastellu-Etchegorry, Jan G. P. W. Clevers, Leo Goudzwaard, Jan den Ouden, Jan Verbesselt, Martin Herold

**Affiliations:** 1 Laboratory of Geo-Information Science and Remote Sensing, Wageningen University & Research, Droevendaalsesteeg 3, 6708 PB Wageningen, The Netherlands; 2 Image Processing Laboratory (IPL), Parc Científic, Universitat de València, 46980 Paterna, València, Spain; 3 Centre d’Etudes Spatiales de la BIOsphere, Paul Sabatier University, CNES-CNRS, 18 avenue Edouard Belin, CEDEX 4, BPi 2801-31401 Toulouse, France; 4 Forest Ecology and Forest Management Group, Wageningen University & Research, Droevendaalsesteeg 3, 6708 PB Wageningen, The Netherlands

**Keywords:** leaf area index (LAI), Sentinel-2, forest, machine learning, vegetation radiative transfer model, Discrete Anisotropic Radiative Transfer (DART) model

## Abstract

The European Space Agency (ESA)’s Sentinel-2A (S2A) mission is providing time series that allow the characterisation of dynamic vegetation, especially when combined with the National Aeronautics and Space Administration (NASA)/United States Geological Survey (USGS) Landsat 7 (L7) and Landsat 8 (L8) missions. Hybrid retrieval workflows combining non-parametric Machine Learning Regression Algorithms (MLRAs) and vegetation Radiative Transfer Models (RTMs) were proposed as fast and accurate methods to infer biophysical parameters such as Leaf Area Index (LAI) from these data streams. However, the exact design of optimal retrieval workflows is rarely discussed. In this study, the impact of five retrieval workflow features on LAI prediction performance of MultiSpectral Instrument (MSI), Enhanced Thematic Mapper Plus (ETM+) and Operational Land Imager (OLI) observations was analysed over a Dutch beech forest site for a one-year period. The retrieval workflow features were the (1) addition of prior knowledge of leaf chemistry (two alternatives), (2) the choice of RTM (two alternatives), (3) the addition of Gaussian noise to RTM produced training data (four and five alternatives), (4) possibility of using Sun Zenith Angle (SZA) as an additional MLRA training feature (two alternatives), and (5) the choice of MLRA (six alternatives). The features were varied in a full grid resulting in 960 inversion models in order to find the overall impact on performance as well as possible interactions among the features. A combination of a Terrestrial Laser Scanning (TLS) time series with litter-trap derived LAI served as independent validation. The addition of absolute noise had the most significant impact on prediction performance. It improved the median prediction Root Mean Square Error (RMSE) by 1.08 m^2^ m^−2^ when 5 % noise was added compared to inversions with 0 % absolute noise. The choice of the MLRA was second most important in terms of median prediction performance, which differed by 0.52 m^2^ m^−2^ between the best and worst model. The best inversion model achieved an RMSE of 0.91 m^2^ m^−2^ and explained 84.9% of the variance of the reference time series. The results underline the need to explicitly describe the used noise model in future studies. Similar studies should be conducted in other study areas, both forest and crop systems, in order to test the noise model as an integral part of hybrid retrieval workflows.

## 1 Introduction

Vegetation represents a primary component in Earth’s terrestrial carbon cycle, with respect to its role both as a source of CO_2_ through respiration and as a sink of CO_2_ through photosynthesis [1]. Its photosynthetic capacity is a function of available leaf area, which can be quantified in terms of Leaf Area Index (LAI). LAI is the leaf area per horizontally projected ground area [2]. It was acknowledged as an Essential Climate Variable (ECV) with high priority in the European Space Agency (ESA)’s Copernicus program [3] and is a focus area of the Committee on Earth Observing Satellites (CEOS) Working Group on Calibration and Validation (WGCV) Land Product Validation (LPV) subgroup [4].

Multi-spectral Earth observation data are sensitive to the amount of canopy foliage, primarily in the NIR and SWIR spectral region [5]. In the NIR, the leaf mesophyll with its enclosed air and air–tissue interfaces results in high reflectance. In the SWIR, the water contained in the leaves leads to high reflectance. Denser leaf layers increase these effects on the canopy level. In this context, the multi-spectral Landsat missions and especially its latest satellite Landsat 8 [6] have shown potential to estimate crop LAI [7–9], forest LAI [10] and in combination with Radiative Transfer Models (RTMs)’ generic LAI [11]. However, the missions’ orbits defines the revisit time as 16 days, which may be insufficient to track fast vegetation changes such as spring leaf flush. The Sentinel-2 mission was awaited for the purpose of estimation of biophysical parameters such as LAI. Its higher spatial resolution, higher revisit frequency and additional red edge bands are emphasised as potentials for performance advances, when compared to the Landsat missions. Agricultural testing campaigns such as SEN3Exp and SicilyS2EVAL offered measurements to explore opportunities for estimating vegetation parameters with spectral observations from Sentinel-2 [12–15]. The SPOT5 Take5 satellite campaign delivered a dataset with a 5-day revisit time to explore the temporal domain [16]. However, the domain of forest remote sensing has not seen this extent of targeted preparation campaigns. Nevertheless, approaches are advancing to make use of the Sentinel-2 open data in terms of biophysical parameter estimation [17,18]. All these campaigns and connected studies underlined that Sentinel-2 has high potential for estimation of LAI and Chlorophyll a and b *(C_a_b)* in diverse canopies.

In parallel with the advances in sensor technology and data availability, Machine Learning Regression Algorithms (MLRAs) were introduced as retrieval techniques [14,19–23]. Their main advantages are their ability to map the nonlinear relationship between canopy parameters and the reflectance signal, and their fast mapping speed, compared to look-up tables [23]. Especially Gaussian Process Regression (GPR)—a kernel-based MLRA—showed good results in terms of prediction accuracy and could achieve the 10% precision for *C_ab_* retrieval required by the Global Climate Observing System (GCOS) [14]. Like traditional retrieval techniques, MLRAs require a database of biophysical parameters, and their associated reflectance signal to learn the mapping between spectral bands and biophysical parameters. This database can originate from field observations, but also from vegetation RTMs. RTMs simulate the spectral properties of vegetation based on a limited set of biophysical parameters and the laws of radiative transfer, which makes them universally applicable. Verrelst et al. [23] conclude that MLRAs and RTMs combined in training workflows have a potential for implementation in operational processing chains for retrieving biophysical parameters. These workflows are referred to as hybrid training workflows.

Turbid medium RTMs, which approximate the vegetation canopy as one homogeneous layer, dominate inversion workflows (e.g., [19,24]). Among these RTMs, PROSAIL—a combination of the PROSPECT leaf and the SAIL canopy model [5]—is one of the most widely used. The main reasons for the use of turbid medium models are their fast processing speed and the low number of input parameters. However, PROSAIL does not agree well with geometrically explicit RTMs in the case of heterogeneous scenes, i.e., scenes where natural objects such as trees are explicitly modelled [25]. As modelling capabilities have outrun means to collect ground truth, a final evaluation of the differences remains open [26]. Apart from this, so-called emulators make it possible to build fast surrogates for complex models [27]. For that, a complex physical model is replaced with a statistical learning model that was trained on input–output combinations of the physical model. This makes it practically possible to exploit heterogeneous RTMs in operational contexts.

In the context of hybrid training workflows, addition of noise to the RTM generated spectral data has been a common practice [19,28–30]. This is typically achieved by adding an absolute term or multiplying with a multiplicative term across the RTM generated spectral bands following a Gaussian distribution. The addition of noise has multiple purposes: it simulates errors of radiometric calibration, atmospheric noise and residuals from the atmospheric correction, but to some extent also bridges between the simplified representation of the RTM and the actual radiometric behaviour of the canopy [19]. Generally, noise prevents the inverse model from over-fitting on the training database. However, an accurate quantification of all error terms in the sensing process remains difficult [19]. Additionally, the quantification of the optimal noise level to be added has not been attempted for hybrid workflows yet.

Considering these developments together—decametric observations from Sentinel-2 and Landsat, fast mapping with MLRA algorithms and fast radiative transfer modelling with emulators—a decametric LAI product would be possible. Such a product would offer better opportunities to monitor ecosystems in fragmented landscapes than comparable products on hectometric scale such as the MODerate-resolution Imaging Spectroradiometer (MODIS) [31] and CYCLOPES [19] LAI products. However, the discrete implementation of hybrid learning processing chains requires many decisions to be taken on—for example, the used RTM and MLRA. Often implemented, but rarely discussed in depth so far, is the addition of noise to the synthetic spectral data.

The aim of this study was to compare different hybrid retrieval workflows that make combined use of Sentinel-2A (S2A) MultiSpectral Instrument (MSI), Landsat 7 (L7) Enhanced Thematic Mapper Plus (ETM+) and Landsat 8 (L8) Operational Land Imager (OLI) observations for forest LAI retrieval. Features in the retrieval workflow were altered in a fully-factorised way to explore their effects. The tested features were: (1) addition of prior knowledge of leaf chemistry, (2) the choice of RTM, (3) the addition of Gaussian noise to RTM produced training data, (4) possibility of using Sun Zenith Angle (SZA) as an additional MLRA training feature, and (5) the choice of MLRA. Performance statistics for the realisations were derived to identify the relevance of the features with respect to prediction performance. Apart from this, features were analysed in terms of their first degree interactions with other features.

## 2 Data

### 2.1 Study Site

**Figure 1:**
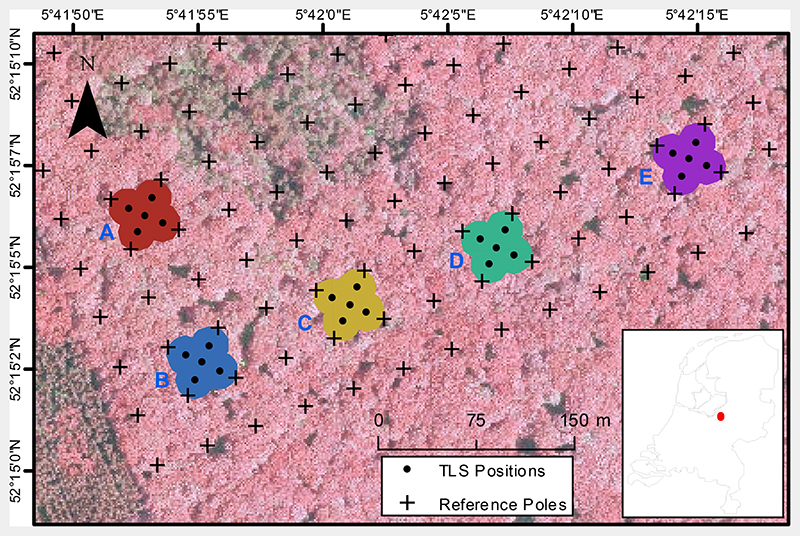
Speulderbos study site with TLS scan positions and polygons representing the five plots. Background image is an airborne false-colour composite of 2013. The inset shows the location of the study site within the Netherlands.

This study focussed on the Speulderbos Fiducial Reference site in the Veluwe forest area (N 52° 15.15′ E 5°42.00′), The Netherlands [32,33] (www.wur.eu/fbprv). In an earlier forest inventory, the site was marked with a wooden pole grid with 40 m spacing, which was geo-located with a Leica™ Robotic Total Station (Leica Geosystems AG, Heerbrugg, Switzerland) and triangulation. This grid served to define the five plots A to E used as validation for this study (Figure 1). The plot locations were chosen with a distance of at least 80 m between them. This distance is four times the image registration error according to S2A mission definition [34].

The stand is predominantly composed of European beech *(Fagus sylvatica)*. A few specimens of pedunculate oak *(Quercus robur)* and sessile oak *(Quercus petraea)* can be found as well. In the understorey, few specimens of evergreen European holly *(Ilex aquifolium)* can be found with heights of <7m in plots A, B, and E. The stand was created as a plantation in 1835 and left unmanaged from then on, so that dominant trees are of even age. Recruitment took only place in canopy openings caused by falling trees as was the case in plot D. This was reflected by the total number of stems, which was 1059 ha^−1^ in plot D compared to 280, 250, 280 and 202 ha^−1^ in plots A, B, C and E, respectively, as determined in a forest inventory in 2013/2014. Basal area was 43.0, 42.5, 31.4,34.8 and 37.2 m ha^−1^ for plots A, B, C, D, and E, respectively.

### 2.2 Field Data

#### 2.2.1 Terrestrial Laser Scanning (TLS) Data

During a field campaign in 2016, a TLS time series was acquired that followed the phenological development of the trees in the study site. The five plots were revisited 28 times with a RIEGL VZ-400 scanner (RIEGL LMS GmbH, Horn, Austria). Samples were taken during the growing season, with an increased intensity during leaf flush and senescence. Rain events and wet canopy conditions were avoided as wet canopy elements tend to absorb the laser pulses, thereby introducing a bias in the estimation of gap fraction. A comprehensive list of all sampling dates can be found in Table A1. In each plot, five scan positions were established that have been visited on each field visit, resulting in a total of 25 positions per field visit. The positions were arranged in a star shape with a centre position determined according to the wooden poles and four positions at the corners of imaginary squares with 20 m edge length.

The individual point clouds were processed with the PyLidar package (http://pylidar.org) based on the methodology developed by Calders et al. [35]. This method basically treats the TLS laser pulses as virtual probes similar to point quadrats, which have been proposed for measuring LAI [36]. The underlying assumption is that the canopy is a random medium with a density proportional to its LAI. The canopy density is proportional to the TLS hit probability, while the TLS hit probability is the inverse of the gap fraction. In this aspect, the TLS gap fraction is similar to the gap fraction derived from digital hemispherical photography [37], which is a standard method to measure LAI [38]. The gap fraction was derived by counting pulses that exited the canopy without return versus all pulses fired in the *hinge angle* region, which was approximated by the zenith angle region between 50° and 60°. Terrain correction for vertical profiles as proposed by Calders et al. [39] was not used because only total canopy Plant Area Index (PAI) values were required, which is indifferent to the terrain. Finally, PAI was derived as follows:

(1) $$PAI=-1.1\;\text{log}(P_{gap}(57.5{}^{\circ}))$$

where *P_gap_* (57.5°) is the gap fraction at the hinge angle. PAI is defined as the one sided surface area of all plant material per unit area ground surface [35]. Alternatively, it can be defined as *PAI = LAI* + WAI, where *WAI* is the wood area index. This is different from LAI, which only includes foliage material [35].

#### 2.2.2 Littertrap Data

Additionally, 25 litter traps were installed in the area to directly measure LAI per season. In each plot, five litter traps were positioned close to the TLS sampling points. Their construction was based on recommendations of the Center for Tropical Forest Science (CTFS) Global Forest Carbon Research Initiative Litterfall Monitoring Protocol, version March 2010 (https://forestgeo.si.edu/sites/default/files/litterfall_protocol_2010.pdf). Each trap consisted of a PVC pipe bent to a circle with an area of 0.7 m^2^ and holding a plastic net. The net allows water to drain and prevent decomposition of the litter content. For each trap, the pipe circles were levelled to assure correct surface area and are held in place 1 m over the ground with four wooden poles.

Litter was collected seven times in 2016 over the course of the season on 1 June, 12 August, 7 and 21 October, 11 November, 2 December, as well as on 10 January in 2017 to collect the last fallen leaves of 2016. Sampling dates were chosen to account for the increased litter-fall in autumn. Litter was collected with paper bags; sorted by species and components, i.e., leaves, twigs, and husks; dried for at least 24 h at 65 °C; and weighted. For each litter collection, 100 leaves were randomly sampled, and their area determined with a leaf area meter (Licor Area Meter 3100, LI-COR, Inc., Lincoln, NE, USA). Specific Leaf Area (SLA)—the unit area of leaf per unit mass—was estimated based on these sub-samples [40]. The total LAI per trap for the whole season was then inferred from the total collected dry leaf weights taking into account the litter trap surface area of 0.79 m^2^. Plot level LAI was estimated as the mean of the single traps.

#### 2.2.3 Leaf Sampling Data

Apart from these canopy structural measurements, the leaf chemistry was monitored over the course of the year by inversion of leaf spectral samples. For this purpose, two beech trees between plot A and B were rigged with ropes and climbed four times at different points of leaf development. On each tree, five branches were cut off at each sampling event from near the crown top. From each branch, five leaves were sampled randomly when no differences in development stage were visible. Especially during the last sampling event, the leaves showed different stages of senescence. In that case, the leaves were sampled to represent the abundance of the respective senescence stage on the branch. Leaf reflectance spectra were acquired with a Fieldspec Pro 3 (ASD Incorporated, Boulder, CO, USA) equipped with an integration sphere. Additionally, the same leaves were sampled with a Minolta SPAD-500 chlorophyll meter (Spectrum Technologies, Inc., Plainfield, IL, USA). For this, four SPAD measurements per leaf were averaged. The sampling resulted in a total of 173 reflectance spectra.

### 2.3 Satellite Data

#### 2.3.1 Sentinel-2A MSI

S2A MSI was primarily designed for land cover and disaster monitoring, but also for retrieval of biophysical parameters such as Fraction Absorbed Photosynthetically Active Radiation (FAPAR), LAI and Fractional vegetation cover (FCover) [34]. Operational products incorporate the Top Of Atmosphere (TOA) Bidirectional Reflectance Factor (BRF) and recently the Surface Reflectance (SR) BRF. Table 1 gives an overview of the MSI spectral bands.

S2A MSI TOA BRF products for tile T31UFT were downloaded from the Copernicus Open Access Hub (https://scihub.copernicus.eu/) for the period of January 2016 until December 2016. The relative orbits R008 and R051 both include the Speulderbos site, thereby doubling the number of observations compared to single orbit observation. TOA BRF products were further processed with sen2cor 2.4.0 (http://step.esa.int/main/third-party-plugins-2/sen2cor/) to derive SR BRF products. During further processing, 60 m bands (B01, B09, B10) were excluded because they are heavily affected by atmospheric conditions and, therefore, surface reflectance products were not provided for these bands. Cloud and quality screening was performed manually under consideration of the scene classification delivered with sen2cor. For this, Normalised Difference Vegetation Index (NDVI) time series for the extracted observations were inspected. Potentially cloud free scenes were identified as high NDVI values in the time series and, therefore, those images were accepted for further processing.

**Figure 2:**
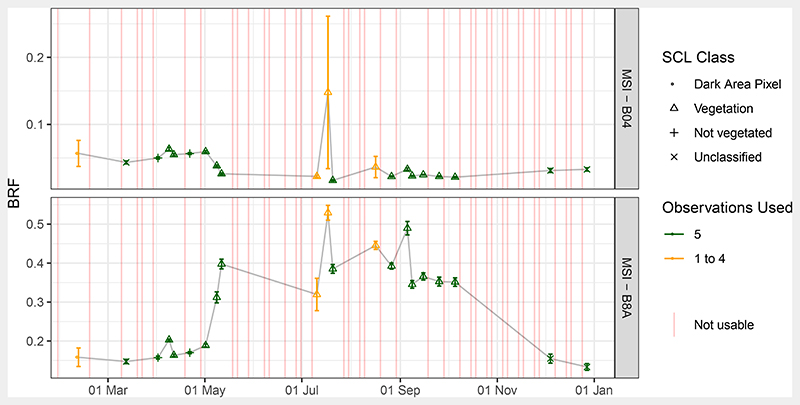
Observed BRFs for S2A MSI red and NIR band over the year 2016 for the five Speulderbos plots (see Figure 1). Points represent average BRF over the five plots, error bars one standard deviation. Colour codes the number of plots for which the observations were useful (clear sky). SCL class refers to the mode of all *Scene Classification* (SCL) given by sen2cor over the five plots. Observations used refers to how many of the BRF of the five plots were not affected by clouds and used for analysis.

Of the 64 dates in 2016 when MSI observations of the Speulderbos site were available, 21 dates yielded usable observations, which is 32.8% of all. Figure 2 shows the bands B04 and B8A, which are the red and NIR spectral bands (Table 1) and hold most information on change in canopy characteristics. The automatic scene classification could identify most of the cloud affected conditions with an accuracy of 91.6%. It should be noted that all observations that were not clouds were further processed, no matter their assigned Scene Classification (SCL) class. Overall, the time series depicted the start of season in April and May: the red reflectance decreased over all plots from 0.059 ± 0.003 on May 1 to 0.022 ± 0.001 on May 11 due to absorption by chlorophyll. At the same time, reflectance in band B8A increased from 0.188 ± 0.008 to 0.406 ± 0.014, which can be attributed to the leaves’ characteristic scattering behaviour. During late summer, the overall temporal course remained stable with a slightly decreasing trend. This trend could also be observed in the validation time series (Figure 6). An exception was 17 July, when B8A reflectance jumped to 0.526 ± 0.023. There were no clouds over the plots on that day, but clouds close by probably caused adjacency effects.

#### 2.3.2 Landsat ETM+ and OLI

Similar to S2A, atmospherically corrected data are available from the Landsat Archive. For this study, L7 ETM+ and L8 OLI SR BRF products at Worldwide Reference System (WRS) row 24 and WRS path 197 and 198 were obtained as on-demand download products provided by the United States Geological Survey (USGS) Earth Resources Observation and Science (EROS) Center Science Processing Architecture (ESPA) On Demand Interface (https://landsat.usgs.gov/landsat-surface-reflectance-high-level-data-products). Both Landsat time series profited from two orbits from which Speulderbos can be observed. Clouds and cloud shadows were identified in the same manner as for MSI and with the support of the pixel quality layer delivered with Landsat Collection 1 products.

In order to extract SR from the satellite SR products, the plots needed to be defined in terms of geo-referenced polygons. For this, the circular field of view of the TLS at the five scan positions within each plot (Figure 1) was considered. Each position was buffered with a circle of 14.4 m radius, which corresponds to the top of canopy of the approximated canopy height of 25 m and maximum observation angle 60° of the TLS. The combined area of the circles represented the plots. The square SR product pixels did not correspond exactly to the polygonal definitions of our plots. Therefore, pixels overlaying each plot were weighted according to their overlap area with the respective plot polygon in order to estimate the SR of the plot.

**Figure 3:**
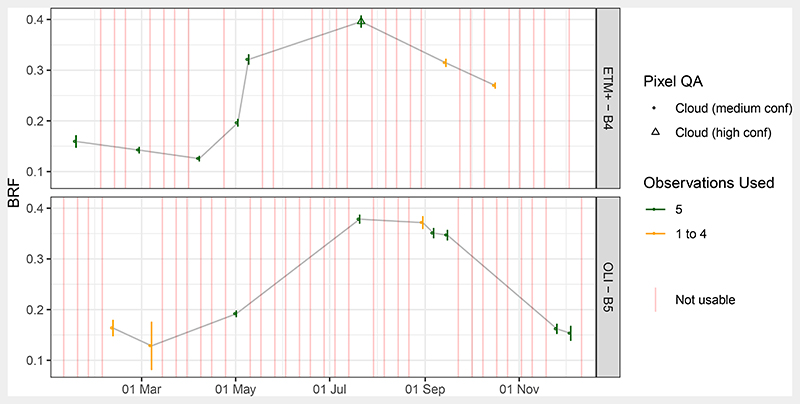
Observed BRFs for Landsat 7 ETM+ and Landsat 8 OLI MSI NIR bands over the year 2016 for the five Speulderbos plots (see Figure 1). Points represent average BRF over the five sites, error bars one standard deviation. Colour codes the number of plots for which the observations were useful (clear sky). Pixel QA refers to the pixel quality bits. Six observations were discarded because they exceeded a reflectance of 1 (undetected clouds). Observations used refer to how many of the BRF of the five plots were not affected by clouds and used for analysis.

In case of the two Landsat missions, 41 and 42 observation dates were available of which 8 (17.1%) and 9 (15.8%) were usable for ETM+ and OLI, respectively (Figure 3). The pixel quality bits for cloud occurence indicated at least medium confidence, which appeared to indicate clear sky conditions after checking the corresponding images. As for MSI, these could mostly be found during the spring green-up and late summer periods. Cloud conditions, represented by bits set to high confidence cloud, were identified with an accuracy of 85.9%.

In September, when multiple observations of MSI and OLI were available, they produced comparable SR BRFs when excluding September 5: average per band BRFs showed differences of less than 5%, which is the S2A mission requirement for SR products. Only L7 ETM+ and S2A MSI showed differences in the NIR of 7.4%, which can be explained by the wider spectral response curve of the ETM+ NIR band 4. These similarities give confidence in comparable behaviour of SR BRFs and to combine observations from these sensors. However, for a detailed comparison the spectral response functions of the sensors and the used atmospheric processors need to be taken into account (e.g., [42]). In fact, for optimal inter-operability, the S2A and Landsat products should be harmonised before combined processing [43].

## 3 Methods

The aim of this study was to modify features—or elements—of a hybrid retrieval workflow for LAI in order to test their impact on the prediction performance. The general order for a hybrid retrieval workflow is as follows [23,44]:

1. A vegetation RTM is run in forward mode to create a database of training samples, i.e., biophysical parameters serve as input for the RTM to predict spectral BRFs. The parameter values are altered to cover multiple canopy conditions.
2. Gaussian noise is added to the spectra to prevent the MLRA from over-fitting and simulate observation noise.
3. Multiple MLRAs are trained on the database to learn the inverse mapping, i.e., from spectral bands to biophysical parameter. Model hyperparameter tuning is performed on a part of the generated database, while the rest is used for testing the trained model.
4. The MLRAs are applied to the observed spectra to predict the biophysical parameter of interest. MLRAs performance is compared.

**Figure 4:**
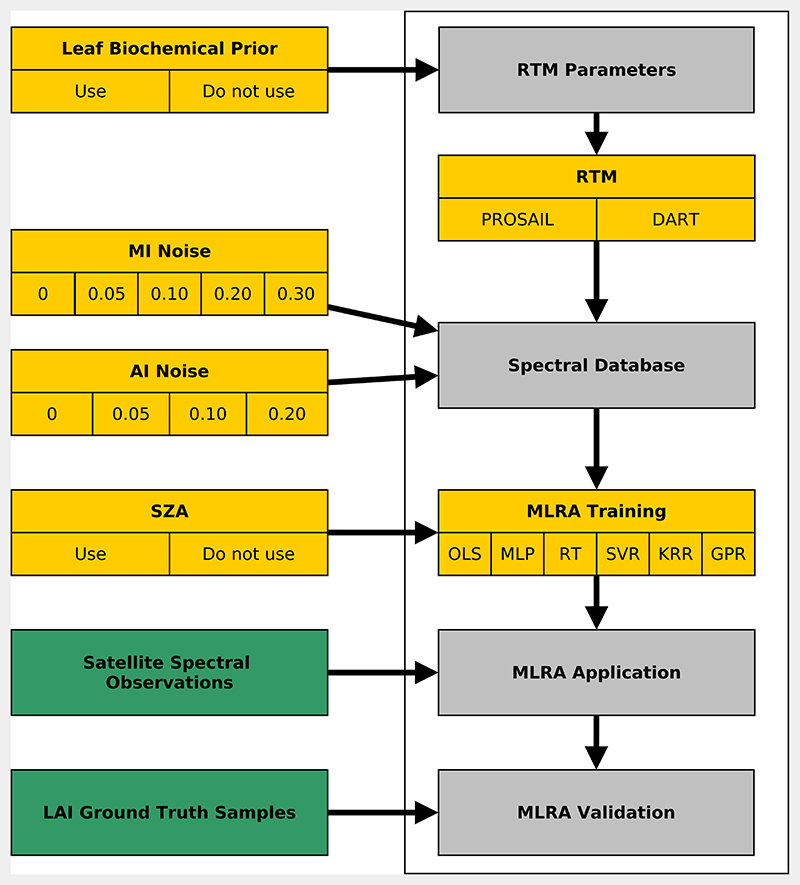
Flowchart of the applied workflow. Varied feature domains have orange background, LAI ground truth green background. For abbreviations, the reader is referred to the text.

The following list gives an overview of the modified features of this study and introduces reference terms under which the feature domains were treated. Figure 4 summarises the workflow and features visually.

- Biochemical Prior: Using leaf biochemical parameters inferred from field spectroscopy observations to restrict the RTM input parameter space (label: *prior knowledge*) versus using a free range (label: *free)* (two alternatives).
- RTM: Two underlying, structurally contrasting RTMs were tested: turbid medium PROSAIL (SAIL 4 coupled with PROSPECT 5) and structurally-explicit Discrete Anisotropic Radiative Transfer (DART) (with PROSPECT 5) (two alternatives).
- Noise scenario: Using multiple noise levels for two types of noise (four and five alternatives).
- SZA: Using the SZA as an additional learning feature (label: SZA) or not (label: *no SZA)* (two alternatives).
- MLRA: Using multiple MLRAs: Ordinary Least Squares (OLS), Multi-Layer Perceptron (MLP), Regression Tree (RT), Support Vector Regression (SVR), Kernel Ridge Regression (KRR), GPR (six alternatives).

Each unique combination of these features is referred to as a *realisation* in the following, while realisations with the same feature were summarised as *ensembles*. For example, a realisation may have used *biochemical prior knowledge*, DART, specific levels of noise, SZA, and OLS. All realisations that implement DART make up the ensemble. All possible combinations of the list above were tested, resulting in 960 realisations. Model training was performed independently for each satellite mission, and model predictions were combined later to form one time series per realisation. A separate per mission performance assessment was not conducted because the effective number of observations varied strongly between the missions and thus would not allow fair comparison.

### 3.1 Leaf Biochemical Parameter Estimation

The spectral signature of vegetation canopies is sensitive to LAI mostly in the NIR, but to a certain extent also in the VIS and red edge, and SWIR [5]. However, the VIS and red edge regions are also strongly affected by *C_ab_*, and the SWIR by leaf water content (*C_w_*) and leaf dry matter content (C_m_). For LAI retrieval, this means having knowledge about leaf biochemistry allows restricting the parameter range that the training database needs to cover, thereby improving the learning of the variations that are caused by LAI. For this reason, a leaf biochemical prior was tested as a possible feature of the training workflow, even though the retrieval of leaf biochemical compounds was not the aim of this study.

For the retrieval of leaf chemical properties, the collected field spectral samples (Section 2.2) were inverted with a gradient descent approach utilising the PROSPECT 5 model as implemented in the R package hsdar (https://cran.r-project.org/web/packages/hsdar) [45,46]. The quasi-Newton method after Byrd et al. [47] that allows box constraints was chosen. Box constraints allow for limiting the searched parameter space to hyper-cubes according to given parameter ranges. The parameter ranges given in Table 2 were chosen as constraints. Results were inspected to identify biochemical compounds whose abundance was stable over the season and could be assumed fixed over the course of the year, or otherwise needed to be treated as variable.

### 3.2 RTMs and Training Database Generation

Two contrasting canopy RTMs were used to represent different levels of canopy complexity. One was PROSAIL, which is a turbid medium model, i.e., it treats the canopy as a homogeneous medium. It is a combination of the PROSPECT leaf and the SAIL canopy bidirectional reflectance model [5]. It has been widely used in the fields of agriculture, plant physiology, and ecology, including estimation of biophysical parameters [16,19,48,49]. In this study, PROSAIL 5B, as implemented in the R package hsdar, was used.

On the other hand, the DART model is a voxel-based flux-tracing model that allows building complex 3D scenes, including vegetation canopies [50,51]. DART contains a PROSPECT module to simulate leaf reflectance and transmission. Applications of DART can be found in the fields of surface energy budget studies [52] and forest biophysical parameter retrieval [53–55], where its advantages of explicitly modelling 3D structure were exploited. DART can be obtained from CESBIO with free licences for publicly funded research and teaching (https://dart.omp.eu).

As both RTMs use PROSPECT 5 as the underlying leaf model, they have many common parameters. Table 2 gives an overview of the used parameter ranges. In the case of *free* realisations, parameters were allowed to vary within the ranges that adopted from the literature [44]. In case of *prior knowledge* realisations, the values were estimated with field spectroscopy as described in Section 3.1. The range of sun zenith angles is based on the geographic location of the Speulderbos site. Since ETM+, OLI and MSI have narrow fields of view of 15°, 15° and 21°, respectively, view zenith angle and relative azimuth angles were assumed to be 0. This was found to be a reasonable assumption for mid and high latitudes [56]. Soil spectra were estimated from MSI barren and winter observations. BRFs were extracted as described above and averaged over all sites. Sensor spectral response curves were approximated as Gaussian with centre wavelength and Full Width at Half Maximum (FWHM) according to published specifications (Table 1). In summary, three models were trained, one for each sensor.

**Figure 5:**
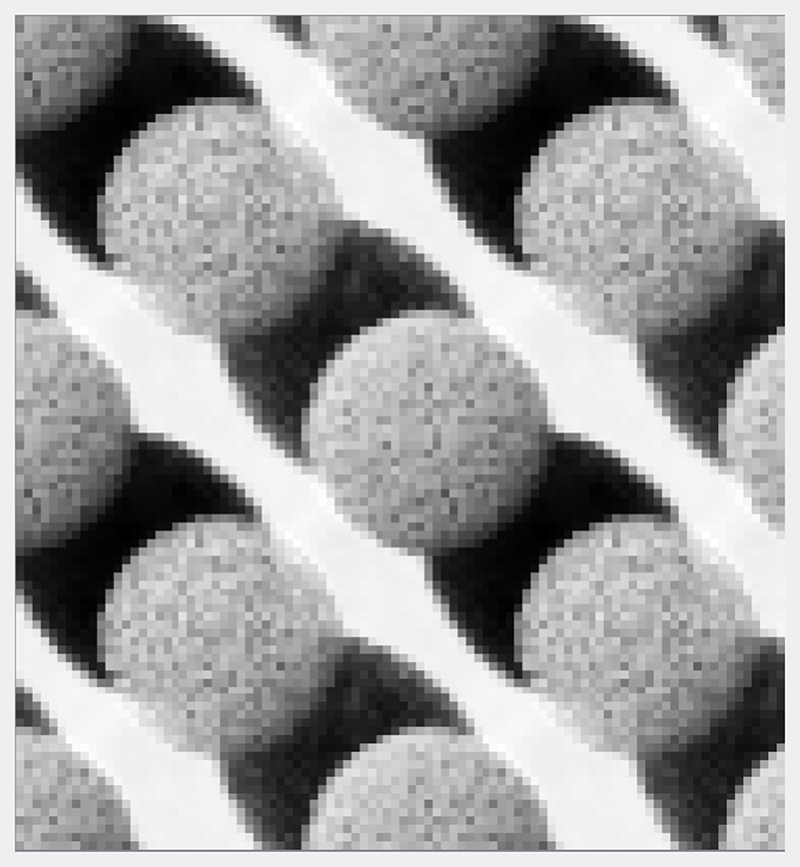
Nadir view of a DART sample scene that is used to represent the heterogeneous canopy. The centre scene with five trees is replicated along the edges. Colour is reflectance with low values black and high values white.

The DART scene was built up of five trees as squared, repetitive scene with 10 m edge length and grid size of 1 m horizontal and 0.5 m vertical cell size (Figure 5). This means that the scene was duplicated along the edges. The trees’ heights and diameters were roughly approximated with TLS point clouds (Table 2, Section 2.2). However, the trees were based on generic forms consisting of 8-faceted stems and ellipsoidal crowns. The stems had a diameter of 0.5 m below and 0.25 m within the crown. The crown leaf volume was simulated as turbid medium cells. TLS was not used to build explicit 3D tree models in order to keep the number of input parameters minimal. For fast computation, an emulator was built to replace actual DART simulations [27]. For this, 2500 samples of DART input parameters were drawn with Latin hypercube sampling [57] according to the *free* option in Table 2. Then, the same MLRAs as for the inversion were trained to predict the single spectral bands of ETM+, OLI and MSI. The best performing MLRA was identified according to the lowest Root Mean Square Error (RMSE) in a five-fold cross-validation.

In total, 2500 parameter samples were drawn with Latin hypercube sampling using uniform distributions to evenly cover the parameter space for all parameters with range specifications. Of these 2500, 30% were modified to represent barren, winter conditions. This means that all leaf chemical parameters and LAI were set to 0.

### 3.3 RTM Sensitivity Analysis

In order to assess the importance of the RTM input parameters (Table 2) on the single spectral outputs, a global sensitivity analysis of the RTMs was conducted. Such an approach also helps to gauge how good input parameters can be estimated from spectral outputs. The approach here generally followed the approach of Verrelst et al. [27] based on Sobol sensitivity indices [58,59] modified by Saltelli et al. [60] and as implemented in the R package sensitivity (https://cran.r-project.org/web/packages/sensitivity). Here, only the total effect indices were considered that describe the sensitivity of the model output to an input parameter and its interactions with other parameters. The sum of the sensitivity indices with respect to all input parameters varies per spectral band output. Therefore, indices were normalised to sum up to 1 to ease comparison across spectral band outputs. Furthermore, only bands of MSI were taken into account because they cover the same spectral domains as ETM+ and OLI (Table 1).

### 3.4 Noise Scenarios

In this study, two types of noise were tested: multiplicative wavelength-independent (MI) and additive wavelength-independent (AI) noise [61]. MI is dependent on the BRF. Its term is larger for NIR compared to red spectral bands for typical vegetation spectral responses. MI and AI were added to the RTM spectral bands:

(2) $$\rho^{\prime }=\rho_{RTM}+\rho_{RTM}\cdot \epsilon_{MI}+\epsilon_{AI}$$

where *ρ^′^* is the noise contaminated spectral band, *ρ_RTM_* is the RTM spectral band output, *∈_MI_* the MI noise term with *∈_MI_ ~ N*(0, *σ_MI_*) and *∈_AI_* the AI noise term with *e_AI_ ~ N*(0, *σ_AI_*). Apart from noise free, realisations with MI noise of 0.05, 0.1, 0.2 and 0.3, and AI noise of 0.05, 0.1 and 0.2 were tested. The 0.05 noise level was motivated by the Sentinel-2 mission requirement of 5% error on SR [34]. The other noise levels were pessimistic variations. However, as mentioned before, the noise term has multiple purposes, so that the mission requirements can only be an indication.

### 3.5 Solar Zenith Angle

Illumination conditions greatly affect the reflectance of canopies [5,62,63]. As the SZA changes over the course of the year, the internal canopy shadowing varies. Furthermore, SZA is an easy to obtain feature, as it is solely a function of location and time. Therefore, SZA was incorporated in the training workflow to test if it improves LAI prediction. SZA was calculated for local overpass times of the respective missions with the R package RAtmosphere ([64], https://cran.r-project.org/web/packages/RAtmosphere). For the respective realisations, it was treated as an extra training feature next to spectral bands.

### 3.6 Machine Learning Regression Algorithms

Studies on MLRA typically test a range of algorithms to explore their respective (dis-)advantages and cross-comparison results. This was adopted in this study as well. All models were trained to predict LAI, while the independent variables depended on the learning realisation. Multi-variate OLS regression was chosen as a benchmark method. For neural networks, the classic MLP was used (e.g., [19]). In particular, this was the implementation of the Stuttgart Neural Network Simulator in the R package RSNNS (https://cran.r-project.org/web/packages/RSNNS). Networks with *n* + 1 neurons in a single hidden layer were trained, where *n* corresponded to the number of independent variables [19]. Random Forest was selected as an RT algorithm [65,66] and used as implemented in the R ranger package (https://cran.r-project.org/web/packages/ranger). The forests were grown with 500 trees.

Furthermore, three kernel-based methods were used. This type of regression methods translates the—possibly nonlinear—regression problem from the parameter space into a higher dimensional feature space, where it can be solved linearly. Kernel functions implement a notion of similarity function. SVR [67], KRR [14] and GPR [68] with Radial Basis Function (RBF) kernels were tested here. The kernel *σ* hyperparameter was estimated with the sigest function from the kernlab package (https://cran.r-project.org/web/packages/kernlab/). Although developed to estimate *σ* for SVR, results in initial tests were promising for KRR and GPR. For further reading on MLRAs in biophysical parameter estimation, the reader is referred to Verrelst et al. [23].

The general workflow for MLRA application typically involves splitting of the feature database into training and validation sets to tune model parameters. Here, five-fold cross-validation was performed during the tuning process. This was based on the training dataset, which held 2500 samples of the RTM-based database. Next, the model performance for the best tuned parameters was evaluated with the test dataset, which held 500 samples of the RTM based database. This set was never seen by the models during training. Finally, the models were applied on the actual sensor observations and compared with the validation dataset (Section 3.7). However, as we inverted observations of three different sensors, in fact, for each realisation, three separate models were trained.

### 3.7 Ensemble Analysis and Validation

In order to analyse how well the MLRAs were able to learn the RTM-produced band-LAI relationships, the test error was evaluated. However, this error represents only the theoretical performance in case the RTM produces true results for the scene and the induced noise properties correspond to the noise of the actual spectral observations.

For validation purposes, the advantages of the TLS time series—i.e., high precision due to independence of illumination conditions [69,70]—was combined with the direct estimation of LAI with the litter-traps. Litter-traps are considered among the most accurate methods in terms of absolute LAI for forest canopies [71]. This approach follows the suggestion of Woodgate et al. [72] to calibrate TLS with other techniques. Specifically, the TLS time series was scaled with the litter-trap total LAI separately for each plot:

(3) $$LAI_{i}=\frac{PAI_{TLS,i}-min\left(PAI_{TLS}\right)}{max\left(PAI_{TLS}\right)}\cdot LAI_{LT}$$

where *LAI_i_* is the LAI at time *i*, *PAI_TLS_* is the TLS derived PAI time series and *LAI_LT_* is the litter-trap LAI. The observations were averaged per plot and linearly interpolated to obtain a continuous time series.

For each realisation, the time series of predicted LAI was compared with this validation time series. The performance metric was the RMSE:

(4) $$RMSE=\sqrt{\frac{1}{t}\sum_{i=1}^{t}{\left(LAI_{realisation,i}-LAI_{valid,i}\right)}^{2}}$$

where *t* is the length of the time series, and *LAI_realisation,i_* and *LAI_valid,i_* the realisation and validation LAI at time *i*, respectively.

## 4 Results and Discussion

### 4.1 Validation Time Series

**Figure 6:**
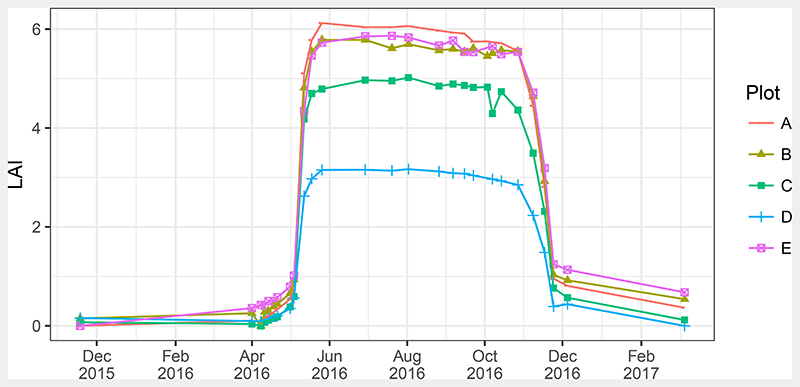
Speulderbos LAI time series for 2016 derived from TLS, points are observations. Lines are linear interpolations. Interpolations for outside of the measurement campaign were performed with the values from the previous and next year, which are added to the graph for clarity.

Figure 6 shows the derived validation time series. The seasonal pattern with a fast spring leaf flush in May and autumn leaf falling in November dominated the temporal behaviour. Calders et al. [35] observed this speed in spring leaf flush for a mixed oak forest in the Netherlands. Maximum LAI of 6.1 m^2^ m^−2^ was reached on 26 May in plot A. Plots B and E showed LAI values just below 6.0 m^2^ m^−2^. Plot C had a larger gap in its centre so that overall LAI was lower there. Measured LAI for plot D was about two units lower than for the other plots with a peak of 3.2 m^2^ m^−2^. This was due to the age composition of this plot, which was dominated by younger trees. The maximum LAI compares well with the results of Leuschner et al. [73], who measured LAI by litter-traps in 23 mature Beech stands in Germany. They found an average LAI of 7.4 m^2^ m^−2^ with a range between 5.6 to 9.5 m^2^ m^−2^.

Another feature is the slow decrease in LAI starting in August that could be observed in all plots. After the 2016 growing season, few brown leaves were still remaining on the trees until new leaves flushed in 2017. Overall, the obtained time series show the expected dynamic behaviour of the canopy during spring and autumn (Figure 6).

With respect to the uncertainty of the validation data, the standard deviation of the mean for the litter-trap samples was calculated as 0.43, 0.25, 0.41, 0.32 and 0.24 m^2^ m^−2^ for plots A to E, respectively. GCOS specified an accuracy of 0.5 m^2^ m^−2^ as a target for LAI products for local and regional applications [38]. However, this is the requirement for the final LAI products, so that the achieved uncertainties are rather high for a validation measurement. This affected especially the realisation evaluations in terms of RMSE.

### 4.2 Leaf Biochemical Parameters’ Retrieval

**Figure 7:**
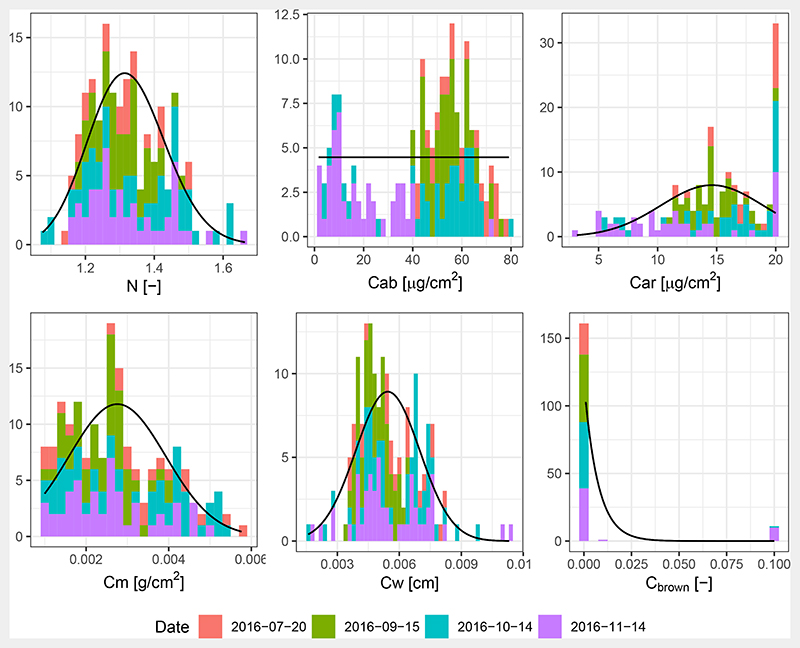
Leaf chemical properties based on PROSPECT inversions of 173 leaf samples. Leaf properties are leaf structure parameter (N), Chlorophyll a and b (*C_ab_*), leaf carotenoid content (Car), leaf dry matter content *(C_m_*), leaf water content *(C_w_*), and leaf brown pigment fraction *(Cb_rown_*). Stacks are coloured by acquisition date. Solid lines are fitted density models. Ordinate axis represents observation counts and modelled counts for the observations and the fitted models, respectively.

The leaf chemistry assessment indicated that some leaf components remained stable over the course of the season, while others showed a dynamic behaviour, resulting in multi-modal distributions (Figure 7). The static ones were the leaf structure parameter (N) parameter, the dry matter content *C_m_*, and to some extent the equivalent water thickness *C_w_*. The mean of the carotenoids remained stable, but its variance was increasing at the last sampling day. *C_ab_* showed clear dynamics, with a strong decrease during the last sampling day. When compared to the readings of the SPAD meter, *C_ab_* retrievals showed a quadratic relationship (Figure A1), which is confirmed by other studies [74,75]. This strong relationship supports the validity of the *C_ab_* retrievals, and thereby the retrieval of the other biochemical constituents since they were inverted simultaneously. On the basis of these results, it was decided to constrain the training with fixed, central values of the N parameter, leaf carotenoid content (Car), *C_w_* and *C_m_*, but vary *C_ab_* and leaf brown pigment fraction (*C_brown_*) as given in Table 2.

### 4.3 RTM Sensitivity

**Figure 8:**
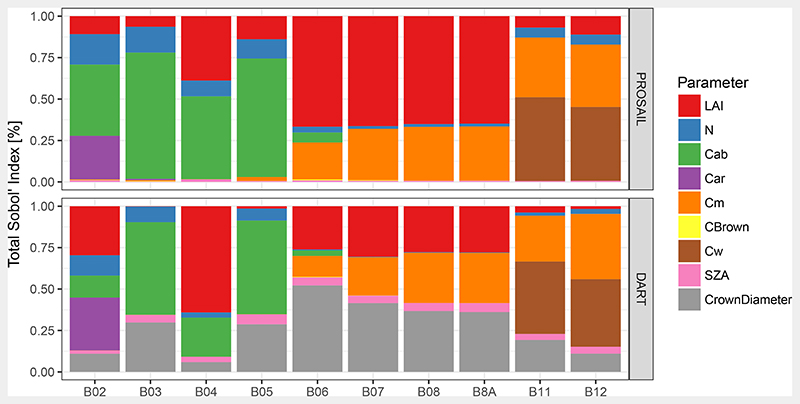
Global sensitivity of S2A MSI spectral bands to PROSAIL and DART input parameters according to Sobol’ Indices. Total Sobol’ Indices were normalised per band to sum up to 1. For band specifications, see Table 1 and for parameters see Table 2.

PROSAIL’s and DART’s sensitivity to their input parameters is depicted in Figure 8. In case of PROSAIL, BRFs in the visible bands were primarily driven by *C_ab_* with a contribution of 74.8 and 49.4% in B03 and B04, respectively. This extended into the first red edge band B05, but strongly decreased to 6.1% at B06. In the red edge and NIR bands, LAI was the most important parameter with a relative contribution of 68.5% for bands B06 to B8A on average. This sensitivity is the reason why LAI retrieval relies on the NIR domain. The SWIR bands were mostly dependent on leaf water content *C_w_*, which had 52.8 and 46.2% contribution in B11 and B12, respectively. These results are in line with those of Jacquemoud et al. [5].

On the other hand, DART’s output sensitivity was dominated by canopy structural parameters (LAI and crown diameter) in the NIR spectral outputs (Figure 8). The contribution of LAI was maximal in B04 with 64.1%, while that of crown diameter was maximal in B06 with 52.2%. Their combined contribution was minimal in B12 with 12.7%. In contrast to PROSAIL, DART showed some sensitivity towards SZA, which was 5.1% on average in bands B05 to B8A. This reflects the effect of shadowing and DART’s vertical heterogeneous character.

### 4.4 Impact of Training Scheme Features on Prediction Performance

This section presents the single inversion workflow features and their role for predicting LAI, starting with a comparison across domains in the following paragraphs. Since the RMSE results were not consistently normal distributed within ensembles, the median was calculated for all realisations that implemented a specific feature. In this way, the feature’s role could be evaluated. It should also be noted that RMSE refers to the validation error. Only in some cases was the training error evaluated, but this is always explicitly mentioned.

Table 3 summarises the effects of all features on the validation RMSE. The 25 and 75% Interquartile Range (IQR) is listed as a quantification for the variability within the ensembles. The feature with the strongest influence on performance in terms of RMSE was the adding of AI noise to the RTM generated spectral outputs. Adding 5% AI noise decreased the median RMSE from 2.33 m^2^ m^−2^ for no noise to 1.25 m^2^ m^−2^. Realisations with 5% AI noise also achieved the overall lowest RMSE. The second-most important feature was the choice of MLRA. Here, realisations varied between RMSE of 2.04 m^2^ m^−2^ for MLP, and RMSE of 1.52 m^2^ m^−2^ for SVR. Restraining the training database with prior information on leaf biochemical contents generally decreased prediction performance.

The following sections present the performance results of all training features in more detail and elaborate on their first order interactions, whereas typically only the strongest interaction is discussed. Interactions were investigated by comparing the respective groups of realisations that implement the features. For example, if feature A has two realisations A1 and A2, and its strongest first order interaction feature B has B1 and B2, all realisations that implement them (A1/B1, A1/B2, A2/B1, A2/B2) were compared to each other. The strongest interaction (feature B in the previous example) was identified as the one that varies performance in terms of validation RMSE the most after the feature under consideration (feature A in the previous example). For example, when the leaf chemical prior was inspected, the feature that produced the largest differences among the realisations was identified as the strongest interaction, which was the RTM feature.

#### 4.4.1 Leaf Biochemical Prior

**Figure 9:**
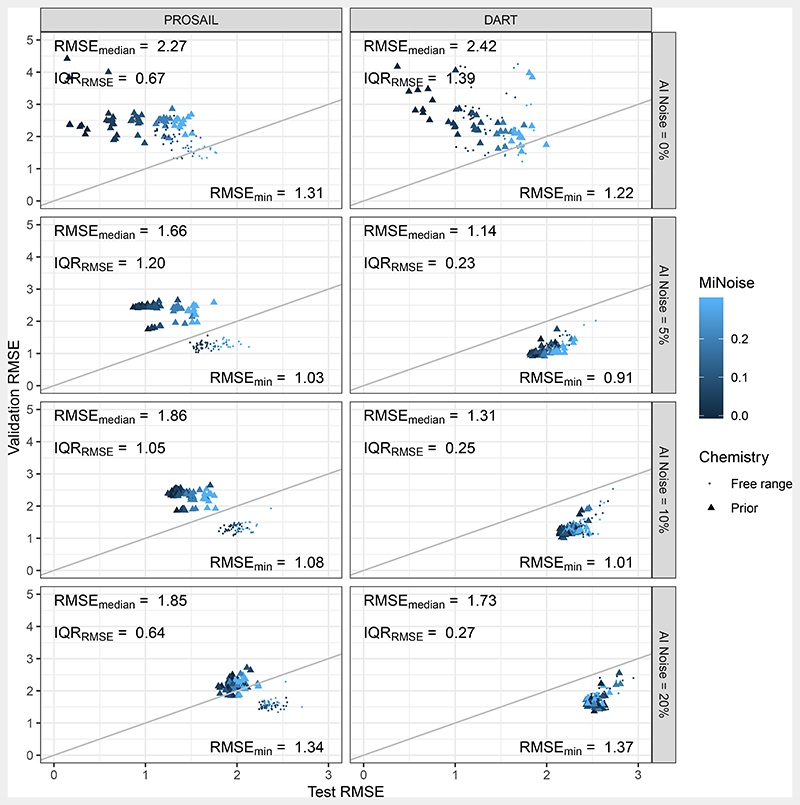
Prediction performance in terms of testing and validation RMSE with dependence on the chosen RTM, AI and MI noise level, and bio-chemical prior. *RMSE_median_*, *IQR_RMSE_* and *RMSE_min_* refer to the validation error in the respective panel cell. Grey line is 1:1 line. In case of the validation results, 14 realisations were trimmed with RMSE larger than 10 m^2^ m^−2^ (all with 0 % AI noise) because they prevented proper display.

Among other information, Figure 9 summarises how the leaf biochemical prior information affected the training results. PROSAIL performance was generally more affected by introducing prior information than DART performance (Figure 9): median RMSE decreased from 1.91 to 1.38 m^2^ m^−2^ when prior information was included in the test data sets, while it increased from 1.43 to 2.37 m^2^ m^−2^ in case of the validation data. The former can be explained by the sensitivity of PROSAIL to biochemical parameters *C_ab_*, *C_m_* and *C_w_* (Section 4.3). Using prior information effectively decreases the parameter input space that the MLRA has to learn, thereby making it easier for the MLRA. This also made it possible to reach testing RMSE as low as 0.01 m^2^ m^−2^. However, inversion of actual observations was impaired by the constraint of the leaf chemical parameter space. This may be due to the fact that leave chemical properties were not representative for the whole study area, as leaves were only sampled from two beech trees, while oak was also present and environmental conditions may change the chemical composition throughout the study area. Additionally, the way the constraint was implemented—as mean estimates allowing no deviation—may also play a role.

Contrary to this, the DART inversion performance was less sensitive to leaf biochemical parameters (Section 4.3). Median performance in terms of RMSE was similar at 2.29 and 2.16 m^2^ m^−2^ for test data and improved slightly with a decrease in RMSE from 1.55 to 1.46 m^2^ m^−2^ for the validation data set when introducing leaf chemical information. Thus, reducing the input parameter space had a small positive effect during training and validation.

#### 4.4.2 RTM Choice

Among other features, Figure 9 compares the inverse model realisation in terms of their used RTM. Both testing error, i.e., the error based on RTM produced samples, and validation error, i.e., the error based on the validation time series for the Speulderbos site, are shown. Overall, PROSAIL and DART achieved a median test RMSE of 1.63 and 2.21 m^2^ m^−2^, while the validation RMSE was 1.93 and 1.51 m^2^ m^−2^, respectively. The lower performance of DART on the testing samples can be explained with its additional freely varying parameter, the crown diameter. However, with this parameter came the capability to model crown gaps (Figure 5), which led to the better performance in terms of validation RMSE.

Apart from the overall better median RMSE of DART, using this RTM generally decreased spread of error for realisations that implemented this RTM, at least when some AI noise larger than 0% was chosen. For example, the validation RMSE IQR for DART with 0% AI noise was 0.23 m^2^ m^−2^, while it was 1.20 m^2^ m^−2^ for PROSAIL. This means that choosing this DART reduced the importance for a particular choice of the other training features.

There were realisations for which the testing RMSE was disproportionally larger than the validation RMSE. In fact, this was the case for 43.3 and 77.7% of the PROSAIL and DART cases, respectively (Figure 9). Under circumstances where data of the same origin would have been used, this would be unlikely to occur, especially in scenarios with added noise. However, training in this study was based on RTM output and validation on field acquired data.

#### 4.4.3 Noise Scenarios

Both the AI and MI noise had different effects on the testing and validation error. Generally testing errors increased with increasing noise—in the case of AI from 1.29 m^2^ m^−2^ for 0 % to 2.46 m^2^ m^−2^ median RMSE for 20% AI noise, and in the case of MI from 1.89 m^2^ m^−2^ for 0% to 2.09 m^2^ m^−2^ median RMSE for 20 % MI noise. This showed the general effect of the noise to blur the relationship between spectral output and associated LAI, and prevent the MLRA to learn the true RTM produced pattern.

AI noise was generally more successful at reducing validation RMSE (Table 3): it decreased median RMSE by 1.08 m^2^ m^−2^ (from 2.33 m^2^ m^−2^ for 0% AI noise to 1.25 m^2^ m^−2^ for 5 % AI noise). MI noise reduced median RMSE only by 0.10 m^2^ m^−2^ (from 1.73 m^2^ m^−2^ for 0 % MI noise to 1.63 m^2^ m^−2^ for 20% MI noise).

Considering AI noise alone, the addition of any in comparison to 0% AI noise strongly changed the distribution of realisations in terms of RMSE (Figure 9). This did not only include the best, but also low performing results. More precisely, AI noise prevented occurrence of very bad results. For the 0% AI noise level, worst performance reached up to 29.29 m^2^ m^−2^ and the 95% quantile lay at 10.4 m^2^ m^−2^, while for 5% these statistics were 2.66 and 2.48 m^2^ m^−2^, respectively. Here, the added noise prevented the MLRA from over-fitting on clean RTM simulations.

Noise was also found an important training workflow element in PROSAIL-Look Up Table (LUT)-based inversions of observations from agricultural crops by Rivera et al. [29] and Verrelst et al. [30]. They added up to 50 and 30% noise to PROSAIL spectra, respectively, but did not specify how the error was implemented. However, the required magnitude of noise corresponds to the MI noise in this study, which was optimal at 20 to 30%. Baret et al. [19] added 0.04 absolute Gaussian white (AI) noise in a global retrieval workflow based on PROSAIL. This is in line with the optimal 5% AI noise in this study. Koetz et al. [28] adopted a wavelength-dependent, relative noise term of maximal 10% in the 444 nm band. Both Koetz et al. [28] and Baret et al. [19] based their choice for a specific noise level on experience of observation errors, but do not evaluate other possibilities.

Baret et al. [19] argued that the quantification of the error term is difficult, as it includes errors stemming from the radiometric calibration of the sensor, Bidirectional Reflectance Distribution Function (BRDF) normalisation, atmospheric correction, cloud residuals and the RTM representativeness for the actual canopy. Additionally, the interaction of these single terms plus the properties of the used MLRA in an inversion workflow complicates the choice based on experience of errors of the sub-systems. Surely, it is more practical to conduct a sensitivity analysis over validation samples rather than characterising the sensor-inversion system in detail. Moreover, noise terms need to be defined properly to compare them across studies.

#### 4.4.4 SZA

As shown in Table 3, realisations that made use of SZA as an extra training feature achieved overall 0.06 m^2^ m^−2^ smaller median RMSE than realisations that did not include SZA. A Wilcoxon signed-rank test confirmed that the two groups differed significantly (*p <* 0.01). This difference was most prominent with the multiple MLRAs. For example, SVR benefited strongest with a decrease in median RMSE by 0.07m^2^ m^−2^. This can be explained by the richer feature space that the MLRAs had available for learning. Additionally, SZA correlated with the general phenological patterns of the study area with low SZA in summer.

Strategies to include SZA into inversion workflows of multi-temporal observations are not consolidated yet. Koetz et al. [28] computed independent LUTs for different observation dates and consequently SZAs, but they did not investigate the error that would occur if they would not have done so. However, training separate models for multi-temporal time series with multiple observations per year is undesirable due to computational load and the checks that would be necessary to ensure consistent model properties. Campos-Taberner et al. [16] conducted PROSAIL inversions over a full season of L8 OLI, L7 ETM+ and Satellite Pour l’Observation de la Terre (SPOT) High Resolution Geometric (HRG) sensor data and they mention solar-sensor geometry as inputs for PROSAIL, but do not elaborate how these parameters were dealt with in the training database. Baret et al. [19] included SZA as a training feature in their neural network based inversion for the VEGETATION based CYCLOPES LAI product, but again did not evaluate this strategy. However, as the CYCLOPES product has global extents, it spans several degrees of latitude, leading to different regimes of illumination dynamics over the year. Thus, SZA was given importance in past studies and showed some importance here as well, but has not been yet evaluated for global LAI products. At least adding SZA as an additional training feature is an easy implementable option. In addition, SZA is efficient to compute, as only the location and observation time are needed to calculate it. However, interactions with other parameters that change over the time of the year such as soil background and LAI itself also need to be considered.

#### 4.4.5 MLRA

**Figure 10:**
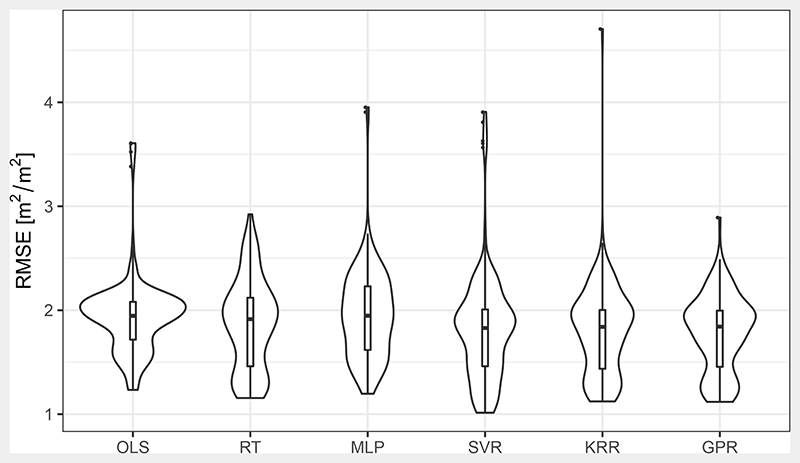
Violin plots [76] of prediction performance for the different MLRAs. It should be noted that 25 realisations were trimmed with RMSE larger than 5.0 m^2^ m^−2^ (18 for OLS and 7 for KRR) because they prevented proper display.

As demonstrated in the discussion of the other training features, the choice for a specific MLRA was not the most important factor affecting validation performance in this study (Table 3). However, studies employing MLRA typically compare various algorithms (e.g., [14,16]). Figure 10 gives an overview over all realisations grouped by their used MLRA. The MLRAs reached best (and median) RMSE of 0.91 (1.57), 0.92 (1.63), 0.93 (1.52), 0.95 (1.60), 1.02 (1.72) and 1.08 (2.04) in case of RT, KRR, SVR, GPR, OLS and MLP, respectively. Hence, even though RT and KRR produced the best, SVR produced the overall best realisations. Maximum differences among the MLRAs in median RMSE of 0.52 m^2^ m^−2^ were found between SVR and MLP realisations. There were also 18 realisations using OLS and seven using KRR that exceeded RMSE of 5 m^2^ m^−2^, all of which were training without noise applied to the training spectral features.

Apart from the MLRA validation, performance processing time is an important property especially for routine and large scale production. The time required for the training of the described realisations in this study was on average 0.03, 7.18, 2.29, 3.17, 125.37 and 123.81 s for the OLS, RT, MLP, SVR, KRR and GPR, respectively. This is in contrast to Verrelst et al. [14] who found KRR and GPR required around the tenth of the time of a neural network or an SVR. However, their models were implemented with Matlab, while this study used R. Additionally, the implementations of RT, SVR, KRR and GPR in this study could make use of parallelisation. Concerning time required for prediction of 10,000 random samples, the models needed 0.08, 0.09, 0.09, 0.15, 0.30 and 0.19 s in the case of OLS, RT, MLP, SVR, KRR and GPR, respectively. Hence, implementation details and optimisation can play a significant role in processing time.

### 4.5 Best Performing Feature Combination

**Figure 11:**
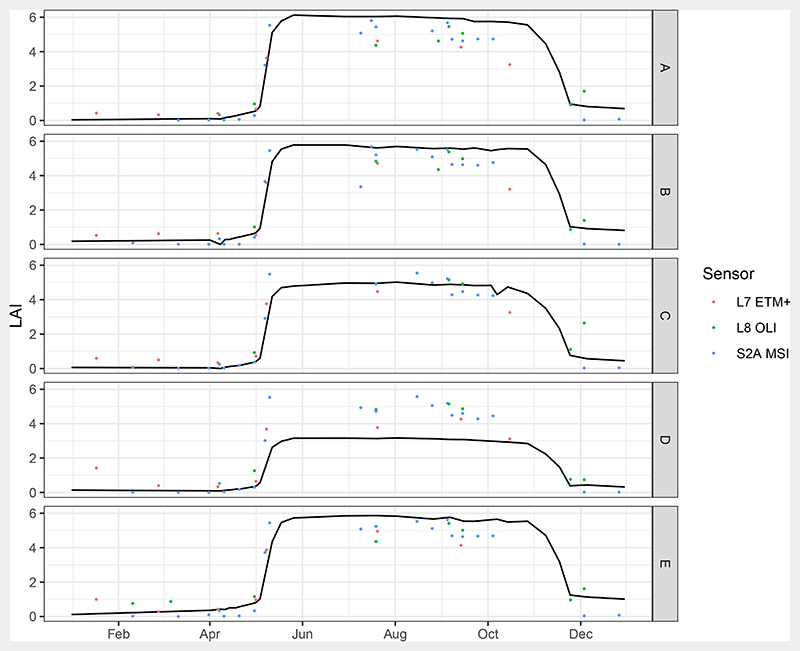
Best performing realisation in terms of RMSE over the 2016 study period. Black solid line is the validation time series.

Figure 11 shows the best performing realisation that reached RMSE of 0.91 m^2^ m^−2^. It was built with an RT based on a DART produced database restricted with prior information from the leaf sampling, 5% AI and 20% MI noise, and with SZA as an additional training feature. Maximum LAI of 5.80 m^2^ m^−2^ was reached in plot A on 17 July. LAI before May 1st was on average (0.27 ± 0.31) m^2^ m^−2^, and thereafter (4.81 ± 0.59) m^2^ m^−2^ until end of October. The predicted LAI explained 84.9% of variation of the reference time series.

In general, these results for a single time series are in the range of previously published results. Schlerf and Atzberger [77] achieved 0.66 m^2^ m^−2^ with a two-band combination chosen from simulated Landsat TM bands over a Norway Spruce site. For beech canopies within the same site, Schlerf and Atzberger [78] report 2.12 m^2^ m^−2^ RMSE with a multi-spectral, near-nadir viewing set-up. Brown et al. [18] obtained a RMSE of 0.47 m^2^ m^−2^ for a beech site in Southern England. All three studies used the INFORM RTM to create the training database [79].

However, as can be observed in Figure 11, the predictions showed different biases for the single plots. In fact, summer LAI was underestimated on average by 0.74 m^2^ m^−2^ in plots A, B, C and E, and overestimated by 1.53 m^2^ m^−2^ in plot D. The bias in plot D could result from the structure of the plot, which consisted of more young trees compared to the other plots. The position of the litter traps was chosen close to the TLS scan positions, which were possibly not representative for the whole of the plot. Additionally, RMSE as the choice of error metric during retrieval evaluation does not allow the assessment of bias. In fact, additional error metrics would be needed to characterise the bias as well as temporal consistency of the time series.

## 5 Conclusions

The Sentinel-2 mission provides a new science-grade data stream for monitoring of dynamic vegetation behaviour. Together with other missions such as Landsat 7, 8 and future Landsat 9, a characterisation of temporal dynamics in biophysical parameters becomes possible at decametric resolution. Accurate retrieval and a universal processing workflow for potential Sentinel-2 and Landsat harmonised biophysical products remains a challenge. Previous studies identified MLRAs combined with RTMs in hybrid retrieval workflows as a potential solution. This study investigated the impact of multiple properties of such workflows on the retrieval performance for LAI over a Dutch beech forest site.

Addition of AI noise on the RTM spectral database was found to be most important for prediction performance with a difference of 1.09 m^2^ m^−2^ in median RMSE compared to no noise. A level of 5% was optimal in this study. On the other hand, MI noise showed less improvements. Added noise helps the MLRAs to generalise and prevent over-fitting on the pure RTM output. Previous studies did not investigate the effect of different noise definitions and some did not report precisely how noise was defined. With respect to its importance, a clear definition and careful sensitivity analysis should be paramount for future studies.

The choice for the heterogeneous DART RTM in comparison with the turbid medium PROSAIL model resulted in a median RMSE difference of 0.42 m^2^ m^−2^. An additional advantage of DART in this study was the lower spread of performance of other inversion workflow features, i.e., DART led to more consistent inversion workflows. Apart from this, care must be taken when using the PROSAIL model with biochemical prior information, as PROSAIL is very sensitive to this. The choice of a specific MLRA was found to be less critical in terms of prediction performance. However, MLRAs varied significantly in run-time, also depending on the implementation and code optimisation. When choosing a particular MLRA, these secondary benefits should be weighted together with the expected accuracy.

## Supplementary Material

Appendix A

## Figures and Tables

**Table 1:** Spectral band specifications for bands and missions used in this study (band centres and widths in nm), bands used for atmospheric correction were omitted [34,41].

Domain	Landsat 7 ETM+			Landsat 8 OLI			Sentinel-2A MSI		
	Name	Center	Width	Name	Center	Width	Name	Center	Width
VIS	B1	485	70	B2	482	60	B2	490	65
	B2	560	80	B3	561	57	B3	560	35
	B3	660	60	B4	654	37	B4	665	30
NIR	B4	835	130	B5	864	30	B5	705	15
							B6	740	15
							B7	783	20
							B8	842	115
							B8A	865	20
SWIR	B5	1650	200	B6	1608	84	B11	1610	90
	B7	2220	260	B7	2200	187	B12	2190	180

**Table 2:** RTM parameters with their symbols, units, ranges (in case of *free* realisations) and best-estimate values (in case of *prior knowledge* realisations; based on PROSPECT inversion of sampled leaf spectra, Section 3.1). The best-estimate values were used for the *prior knowledge* realisations.

	Model Parameter	Unit	Free	Best Estimate
Leaf parameters: PROSPECT-5B				
N	Leaf structure index	-	1–2.5	1.27
*C_ab_*	Leaf chlorophyll content	μg cm ^−2^	0–80	-
Car	Leaf carotenoid content	μg cm^−2^	0–20	8.60
*C_m_*	Leaf dry matter content	g cm^−2^	0.001–0.025	0.00263
*C_w_*	Leaf equivalent water thickness	cm	0.002–0.025	0.0053
*C_brown_*	Brown pigment fraction	-	0–1	-
Canopy parameters: SAIL4 and DART				
*LAI*	Leaf area index	_m_^2^_m_^−2^	0–8	0–8
*θ_s_*	Sun zenith angle	°	27.5–80	27.5–80
*θ_o_*	View zenith angle	°	0	0
*ϕ*	Sun-sensor azimuth angle	°	0	0
*LAD*	Leaf angle distribution	-	Plagiophile	Plagiophile
Canopy parameters: SAIL4				
*α_soil_*	Soil wet/dry factor	-	0	0
*hspot*	Hot spot parameter	-	0	0
Canopy parameters: DART				
*TreeHeight*	Tree height	m	20	-
*Crown Diameter*	Tree crown diameter	m	5–9	-
*Crown Height*	Tree crown height	m	7	-

**Table 3:** Validation median RMSE and Interquartile Range (IQR) (25 to 75%) for training features. ΔRMSE refers to the difference to the best feature per group.

Feature	Realisation	Median RMSE	ΔRMSE	RMSE IQR
Leaf chemical prior	Free range	1.47	—	0.49
	Prior	2.10	0.63	0.96
RTM	PROSAIL	1.93	0.42	0.95
	DART	1.51	—	0.69
MI Noise	0%	1.73	0.10	1.12
	5%	1.70	0.07	1.08
	10%	1.70	0.07	1.01
	20%	1.63	—	0.84
	30%	1.63	—	0.73
AI Noise	0%	2.33	1.08	0.80
	5%	1.25	—	0.67
	10%	1.38	0.13	0.68
	20%	1.74	0.49	0.51
SZA	Without SZA	1.69	0.06	0.97
	With SZA	1.63	—	0.95
MLRA	OLS	1.72	0.20	0.54
	MLP	2.04	0.52	0.88
	RT	1.57	0.05	1.04
	SVR	1.52	—	1.06
	KRR	1.63	0.11	1.06
	GPR	1.60	0.08	0.98
