# Supplementary material for: Assessment of Workflow Feature Selection on Forest LAI Prediction with Sentinel-2A MSI, Landsat 7 ETM+ and Landsat 8 OLI
Source: Remote Sens (Basel). Author manuscript; Available in PMC 2022 Sep 7. (PMC7613362; doi:10.3390/rs12060915)
Supplement: Appendix A [file EMS152651-supplement-Appendix_A.pdf]

**Author Contributions:** Conceptualization, B.B., J.V. (Jochem Verrelst), J.G.P.W.C.; methodology, B.B., J.V. (Jochem Verrelst), J.-P.G.-E., J.G.P.W.C.; formal analysis, B.B., J.-P.G.-E., L.G., J.d.O.; data curation, B.B., L.G.; writing–original draft preparation, B.B., J.G.P.W.C.; writing–review and editing, B.B., J.V. (Jochem Verrelst), J.-P.G.-E., J.G.P.W.C., J.V. (Jan Verbesselt); visualization, B.B.; supervision, J.G.P.W.C., J.V. (Jan Verbesselt), M.H.; project administration, M.H.; funding acquisition, M.H. All authors have read and agreed to the published version of the manuscript.

**Funding:** This research was funded by the IDEAS+ and QA4EO contracts funded by ESA-ESRIN. Jochem Verrelst was funded by the European Research Council (ERC) under the ERC-2017-STG SENTIFLEX project (grant agreement 755617).

**Acknowledgments:** Data analysis was supported by the Research and User Support (RUS) Service. The RUS Service is funded by the European Commission, managed by the European Space Agency, and operated by CSSI and its partners. The authors thank the Dutch Forestry Service (Staatsbosbeheer) for granting access to the site, and Devis Tuia and Benjamin Kellenberger for helpful discussions on machine learning. We thank two anonymous reviewers for their valuable comments.

**Conflicts of Interest:** The authors declare no conflict of interest. The funders had no role in the design of the study; in the collection, analyses, or interpretation of data; in the writing of the manuscript, or in the decision to publish the results.

## Appendix A

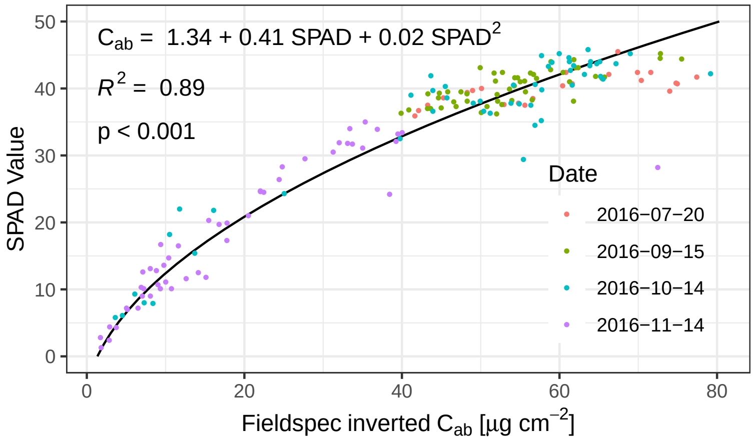


**Figure A1.** Comparison of Fieldspec inverted $C_{ab}$ with SPAD meter value taken from the same leaf samples. Solid line, $R^2$, $p$-value and formula correspond to the quadratic fit. Dates correspond to the leaf sampling dates.

**Table A1.** List of TLS and satellite sampling dates.

| TLS | Landsat 7 | Landsat 8 | Sentinel-2A |
|---|---|---|---|
| 2015-11-18 | 2016-01-18 | 2016-02-11 | 2016-02-11 |
| 2016-04-01 | 2016-02-28 | 2016-03-07 | 2016-03-12 |
| 2016-04-08 | 2016-04-07 | 2016-05-01 | 2016-04-01 |
| 2016-04-11 | 2016-05-02 | 2016-07-20 | 2016-04-08 |
| 2016-04-14 | 2016-05-09 | 2016-08-30 | 2016-04-11 |
| 2016-04-18 | 2016-07-21 | 2016-09-06 | 2016-04-21 |
| 2016-04-21 | 2016-09-14 | 2016-09-15 | 2016-05-01 |
| 2016-05-01 | 2016-10-16 | 2016-11-25 | 2016-05-08 |
| 2016-05-04 | | 2016-12-04 | 2016-05-11 |
| 2016-05-12 | | | 2016-07-10 |
| 2016-05-18 | | | 2016-07-17 |
| 2016-05-26 | | | 2016-07-20 |
| 2016-06-29 | | | 2016-08-16 |
| 2016-07-20 | | | 2016-08-26 |
| 2016-08-02 | | | 2016-09-05 |
| 2016-08-26 | | | 2016-09-08 |
| 2016-09-06 | | | 2016-09-15 |
| 2016-09-15 | | | 2016-09-25 |
| 2016-09-22 | | | 2016-10-05 |
| 2016-10-03 | | | 2016-12-04 |
| 2016-10-07 | | | 2016-12-27 |
| 2016-10-14 | | | |
| 2016-10-27 | | | |
| 2016-11-08 | | | |
| 2016-11-17 | | | |
| 2016-11-24 | | | |
| 2016-12-05 | | | |
| 2017-03-07 | | | |

## References


1. Beer, C.; Reichstein, M.; Tomelleri, E.; Ciais, P.; Jung, M.; Carvalhais, N.; Rödenbeck, C.; Arain, M.A.; Baldocchi, D.; Bonan, G.B.; et al. Terrestrial gross carbon dioxide uptake: Global distribution and covariation with climate. *Science* **2010**, *329*, 834–838. [CrossRef] [PubMed]
2. Chen, J.; Black, T.A. Defining leaf area index for non-flat leaves. *Plant Cell Environ.* **1992**, *15*, 421–429. [CrossRef]
3. Malenovský, Z.; Rott, H.; Cihlar, J.; Schaepman, M.E.; García-Santos, G.; Fernandes, R.; Berger, M. Sentinels for science: Potential of Sentinel-1, -2, and -3 missions for scientific observations of ocean, cryosphere, and land. *Remote Sens. Environ.* **2012**, *120*, 91–101. [CrossRef]
4. Morisette, J.T.; Baret, F.; Privette, J.L.; Myneni, R.B.; Nickeson, J.E.; Garrigues, S.; Shabanov, N.V.; Weiss, M.; Fernandes, R.A.; Leblanc, S.G.; et al. Validation of global moderate-resolution LAI products: A framework proposed within the CEOS land product validation subgroup. *IEEE Trans. Geosci. Remote Sens.* **2006**, *44*, 1804–1814. [CrossRef]
5. Jacquemoud, S.; Verhoef, W.; Baret, F.; Bacour, C.; Zarco-Tejada, P.J.; Asner, G.P.; François, C.; Ustin, S.L. PROSPECT + SAIL models: A review of use for vegetation characterization. *Remote Sens. Environ.* **2009**, *113*, S56–S66. [CrossRef]
6. Roy, D.P.; Wulder, M.A.; Loveland, T.R.; Woodcock, C.E.; Allen, R.G.; Anderson, M.C.; Helder, D.; Irons, J.R.; Johnson, D.M.; Kennedy, R.; et al. Landsat-8: Science and product vision for terrestrial global change research. *Remote Sens. Environ.* **2014**, *145*, 154–172. [CrossRef]
7. Houborg, R.; McCabe, M.; Cescatti, A.; Gao, F.; Schull, M.; Gitelson, A. Joint leaf chlorophyll content and leaf area index retrieval from Landsat data using a regularized model inversion system (REGFLEC). *Remote Sens. Environ.* **2015**, *159*, 203–221. [CrossRef]
